# The Cracking of Al-Mg Alloys Welded by MIG and FSW under Slow Strain Rating

**DOI:** 10.3390/ma16072643

**Published:** 2023-03-27

**Authors:** Krzysztof Dudzik, Mirosław Czechowski

**Affiliations:** Faculty of Marine Engineering, Gdynia Maritime University, 81-256 Gdynia, Poland

**Keywords:** aluminum alloys, welding, MIG, friction stir welding, SSRT, fractography analysis, in situ tensile test

## Abstract

Al-Mg alloys used in the shipbuilding industry were tested. The most commonly used alloy AW 5083 and alloy AW 5059 with higher strength properties were selected. Both native materials and their joints welded by the traditional MIG arc welding method and the friction stir welding (FSW) method were tested. Both methods are approved by classification societies which allow them to be used in construction by the shipbuilding industry. The research was carried out in two stages. The first study was an “in-situ tensile test in SEM”. The surfaces of the deformed specimens’ changes were observed in the vacuum chamber of a Philips XL30 scanning electron microscope. During the tests, the force and elongation of the specimen were recorded. In addition, a chemical analysis of selected precipitates was performed by energy dispersive spectrometry (EDS) using the EDAX adapter. Slip lines were observed on the surface of the tested specimens, which are arranged in bands in the native material and in a disordered manner in the joints welded by MIG and FSW methods. Cracking starts mainly through decohesion at the matrix-precipitate interfaces. In the second stage of the research, slow strain rate testing (SSRT) was carried out in accordance with ISO 7539-7:2005. The tests were carried out on a specially designed test stand, where the FT-5307 strain gauge force sensor with a measuring range of 0–16 kN was used to measure the force value. The PSz 20 transducer with a measuring range of 0–20 mm was also used. The test consisted of subjecting the specimen to increasing deformation with the strain rate έ = 1.6 × 10^−6^ s^−1^ until destruction. The fracture surfaces of the SSRT specimens were subjected to fractographic analysis using a Philips XL-30 scanning electron microscope. The results of fractographic studies after the SSRT test of the native materials and their joints welded by the MIG and FSW methods indicate that the trans-crystalline cracking mechanism is dominant, characterized by ductile fracture, and cracks are initiated at the interfaces of the precipitates with the matrix. The research results show that the plastic deformation increases and micro-cracks develop and merge into a main crack, which, after reaching the critical dimension, rapidly develops, causing the destruction of the material. The fracture micrographs of the specimens of base metals and their joints welded by FSW and MIG after the SSRT test allow us to conclude that the cracking mechanism was trans-crystalline ductile.

## 1. Introduction

Aluminum alloys are widely used in many industries, including those related to transport. One such field is shipbuilding. The increased interest of ship hull constructors in aluminum alloys is dictated primarily by the possibility of a significant reduction in its weight [1,2]. This allows for an increase in the transport capacity of the vessel and an increase in speed or significant savings in fuel consumption. Pure aluminum is characterized by low-strength properties (UTS = 70–120 MPa, YS = 20–40 MPa); therefore, aluminum alloys with other elements are used in practice. In shipbuilding, especially due to the corrosive nature of the environment, mainly Al-Mg alloys (5xxx series) are used. These alloys are currently the most widely used due to their relatively good mechanical properties in service conditions and good weldability.

The most popular is alloy 5083 (AlMg4.5Mn), which is characterized by medium strength properties and good resistance to corrosion, especially stress corrosion [3]. The advantage of this alloy is also its good weldability, using traditional arc welding methods approved by classification societies, such as MIG and TIG. Continuous development in changing the chemical composition of 5xxx alloys resulted in the development of alloy 5059 (AlMg5MnZn) with higher strength properties compared to alloy 5083, while maintaining good weldability and corrosion resistance [4]. Li, W. et al. [5] considered the addition of Li to Al-Mg alloys and the rolling temperature on mechanical properties, reaching strengths of 480 MPa. Stoliara, I.A. et al. [6] also considered the influence of the Li addition on the properties of thin foils made of Al-Mg-Li alloys, achieving a significant improvement in strength properties (up to 33%). Kumar, N. et al. [7] studied the effect of FSP (friction stir processing) treatment temperature on the grain size and thus the mechanical properties of Al-Mg alloys with the addition of Sc. Many researchers, e.g., Tong, M. et al. [8], Li, X. et al. [9], and Ekubaru, Y. et al. [10] considered modifying Al-Mg alloys with Sc and Zr additions in order to improve mechanical properties. Such a great interest in these alloys is dictated by their ultimate tensile strength exceeding 500 MPa. Nokhrin, A. et al. [11] checked the effect of the addition of Sc and Zr on the size of grains and precipitates. Ultimately, they considered the effect of these additives on strength properties and susceptibility to plastic deformation at elevated temperatures. Abrami, M.B. et al. [12] studied the effect of heat treatment on the properties of Al-Mg-Sc-Zr alloys produced by the Laser Powder Bed Fusion method. They also investigated the changes in the concentration and size of precipitates as well as micro-voids. Chen, H. et al. [13] checked the influence of Sc and Zr additions on Al-Mg alloys with regard to their mechanical properties, especially plasticity. In addition, they checked the number and size of precipitates in terms of creating slip planes and thus susceptibility to plastic processing.

Joining aluminum and its alloys by welding methods is difficult due to its specific properties. The main difficulties that occur during the welding of aluminum alloys result from the high chemical affinity of aluminum to oxygen and the formation of hard-melting (2060 °C) Al_2_O_3_ oxide, high thermal conductivity, high expansion of aluminum alloys, high casting shrinkage (the cause of welding deformations and stresses), significant drops in strength at welding temperatures, and loss of alloying elements such as magnesium, zinc, or lithium during welding. The main difficulties related to the welding of aluminum alloys, listed briefly, prompt the search for other methods of joining these materials. Such an alternative for butt joining of sheets is the friction stir welding method [14].

In the FSW (friction stir welding) technology, a tool with a rotating mandrel is used to heat and plasticize the material, fixed in the place where the pressed sheets are joined. After setting the tool in rotation, frictional heat, and plasticization of the sheet material in its immediate vicinity, the entire system slowly moves along the contact line. FSW is a method of welding in the solid state, such as aluminum alloys, copper, stainless steels, and titanium alloys. The technology is still being intensively developed, which allows for its gradual growth in the global industry. An important advantage of this method is the easiness of obtaining joints with high, repeatable properties [4,15]. In addition, it is possible to combine dissimilar materials, which is difficult or even impossible to achieve by other methods. Kim, S. et al. [16] studied the joints of alloy 5083 with alloy 6061, NIU, P. et al. [17] the joints of alloys 2024 and 5083, while Matsuda, T., Ogaki, T., and Hayashi, K. et al. [18] combined alloy 6061 with galvanized steel. Picot, F. et al. [19] described in their paper the results of friction stir welded 5083 alloy sheets with 316 L stainless steel. Chen, Y. et al. [20] described the technology of joining the 2024 aluminum alloy with the TC1 titanium alloy. Shanavas, S. et al. [21] compared alloy 5052 FSW welded joints with TIG welded joints, where they proved that the mechanical properties of FSW joints are better and the joint structure is more homogeneous. Kalashnikova, T. et al. [22] examined the possibility of welding Al-Mg-Sc-Zr alloy sheets with a thickness of up to 35 mm. The joint was made in one pass, which is impossible with traditional arc welding. Similar to other researchers, they obtained the effect of grain fragmentation in the welding zone and an increase in strength properties by about 20% compared to the base material.

The improvement of the FSW joining technology also results in the development of different variants of this technology. One variation is the friction stir spot welding (FSSW) method described by Bagheri, B. et al. [23,24] and Vaneghi, A.H. et al. [25], among others, which is used for joining aluminum alloys with pure copper. Suresh, S. et al. [26] strengthened the alloy 5083 with SiC ceramic particles using this method. A different way to improve the FSW method is the simultaneous use of ultrasonic vibrations. Studies on the impact of the use of the FSW method with ultrasonic vibration for joining alloy 2024 were carried out by Liu, X.C. et al. [27]. Another variant is the vibration-assisted FSW process. As demonstrated by Abbasi, M. et al. [28] and Kim, S. et al. [29], the use of vibration during FSW causes fragmentation of the grains in the stirred zone, which, according to the Hall–Petch equation, increases the strength properties. Fouladi, S. et al. [30] achieved the same results for alloy 5052 regarding the increase in mechanical properties and a slight decrease in the resistance to electrochemical corrosion of FSVW joints compared to FSW. Variations of the FSW method also include friction stir processing (FSP). Mirian Mehrian, S.S. et al. [31] used the FSP method to improve the surface of the Al-Mg alloy both in terms of mechanical properties and resistance to electrochemical corrosion. Liyakat, N.A. and Veeman, D. [32] indicated the FSP method to improve the strength and plastic properties of TIG welded joints on the example of alloy 5052.

The use of aluminum alloys and their joints welded by different methods in various industries requires knowledge of the mechanism of their destruction in a specific environment. Standard mechanical properties tests by static tensile test do not provide sufficient knowledge on the initiation and propagation of cracks under load. One method to understand these phenomena is the in situ tensile test in SEM. Lall, A. et al. [33] investigated austenitic stainless steel, alloy 709, where they showed that the beginning of the cracking process is most often in the place of precipitates. As a result of tensile stress, they are separate from the matrix, creating a void. Li, H. et al. [34] observed the initiation and development of cracks on the surface of a coating applied to alloy 6061 during the bending of samples. They discovered that crack initiation begins with the delamination of the coating from the surface of the base material. Guercio, G. Del et al. [35] described the initiation of cracking at the location of pores in alloy 2024 made by the laser powder bed fusion method.

Slow strain rate testing (SSRT) with subsequent fracture analysis can also determine the nature of the cracking. Aboura, Y et al. [36] and Wang, S. et al. [37] studied the 7xxx alloys, while Li, M. [38] studied the 8xxx aluminum alloy while determining their corrosion resistance. Holroyd, N.J.H. et al. [39] conducted SSRT studies of 5083 alloys where they determined both the development and crack inhibition mechanisms depending on the three indicated types of cracking: type-1 cracking along grain boundaries, type-2 cracking is partly a grain boundary and partly plastic trans-crystalline, and type-3 cracking refers to the final overload failure and comprises shear failure via micro-void coalescence. Popovic, M. et al. [40] used the SSRT method to evaluate the effect of high Mg content as well as heat and plastic treatment in 5xxx alloys on stress corrosion cracking resistance in a corrosive environment. Other researchers conducted studies of FSW welded joints of 5xxx alloys. Torzewski, J. et al. [41] determined the properties of alloy 5083 welded FSW and the influence of the slow strain rate test on crack development. Duan, S. et al. [42] conducted research in a welded joint of alloy 5052, where they determined the type of precipitates and their impact on the development of cracks in the NaCl environment of various concentrations, using the EDS method.

The aim of the work was to determine the effect of slow stretching on deformation development and, consequently, crack initiation and propagation in 5059 and 5083 aluminum alloys and in their joints welded by two methods: the traditional MIG arc welding method and the relatively new, friction stir welding method. The novelty of this article is the comparison of the results of two methods of low-speed tensile testing: in situ tensile test in SEM and slow strain rate testing. The available literature indicates the use of one of the methods to determine the changes occurring in the material during its loading. The use of both methods at different strain rates allowed for a more accurate understanding of the nature of cracking occurring during the stretching process compared to only one method.

## 2. Materials and Methods

Aluminum alloys EN AW-5083 (AlMg4.5Mn) and EN AW-5059 ALUSTAR (AlMg5Mn) produced by the Hoogovens (Velsen-Noord, Netherlands,) were used for the tests. Both alloys were delivered in H321 condition. The chemical composition of the alloys is given in Table 1.

**Figure 1:**
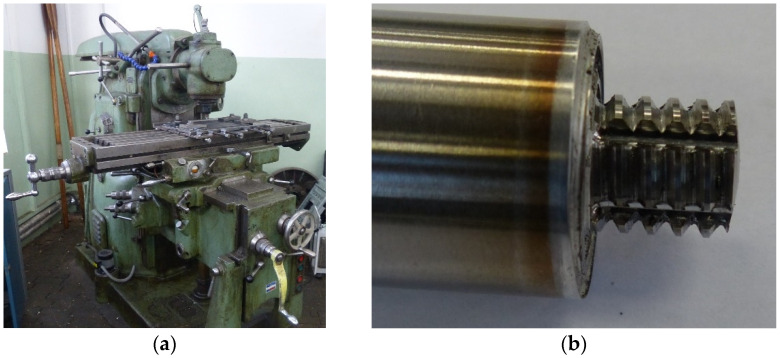
View of (**a**) the FSW welding station and (**b**) the FSW tool.

The thickness of the welded sheets was 12 mm and they were milled on one side to a thickness of 10 mm in the contact parts. Appropriate rigid fastening of the sheets was ensured by the instrumentation of the welding machine, built on the basis of the FWA-31 universal milling machine. The view of the FSW welding station and the tool used is shown in Figure 1.

**Figure 2:**
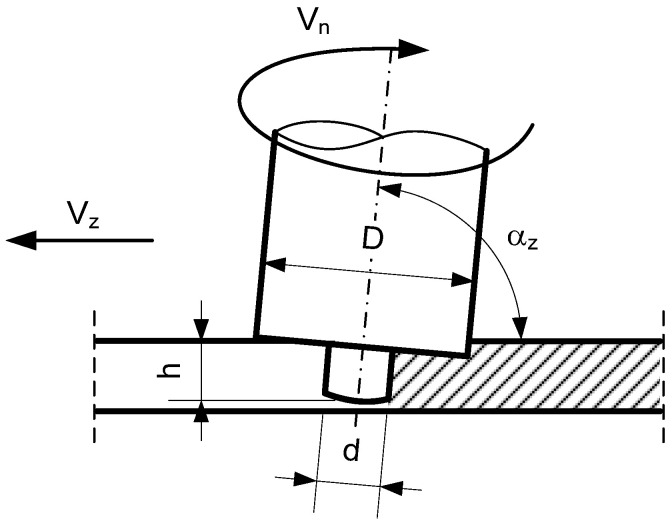
The diagram of FSW.

The contact surfaces of the joined elements were degreased. The sheets were welded on both sides with the same parameters as shown in Table 2. Figure 2 shows an overview diagram of the friction stir welding method. The welding parameters were selected on the basis of previous studies where the criterion of the smallest grains in the stirring zone was used. An additional criterion was the absence of defects and voids in the weld.

For comparison, butt joints welded by the traditional arc method were used. Sheets made of alloys 5059 and 5083 with a thickness of 12 mm were welded using the MIG method. All welded joints were made in one of the companies producing marine structures aluminum, the Wisła Aluminium International Ltd. Shipyard (Gdańsk, Poland). The preparation of welded joints was performed in accordance with the procedures required by the shipbuilding industry. Cutting and bevelling of the edges were prepared by mechanical processing. The sheets were beveled X by the angle of 110° (from the face side) and 60° (from the root side) without a threshold. Before welding, the surfaces of the groove and those in its immediate vicinity were cleaned of oxides using rotating stainless-steel brushes and then degreased with extraction naphtha. Preparation and welding of the joints were carried out in a closed room in order to protect the station against weather conditions. To avoid deformation of the joints, welding was performed in the tooling, and the clamps were released only after the joint had cooled down. The assembly of the joint elements was carried out using tack welds. During welding, the cracked tack welds were cut out due to the possibility of cracks even after careful remelting of the cracked welds. After making the facing seams, the bottom of the face joint was cut out, and then the root layer was laid.

For the welding of alloy 5083, a 5383 alloy wire with a diameter of 1.6 mm was used, while for alloy 5059, a 5183 alloy wire with a diameter of 1.2 mm was used. The electrode wire was etched immediately before welding. The chemical composition of individual alloys used for welding wires is presented in Table 3.

Argon with a purity of 99.99% was used as the shielding gas. The following welding parameters were used: current intensity I = 240–260 A (alloy 5083), I = 210 A (alloy 5059), arc voltage U = 28 V, and argon consumption—25 L/min.

**Figure 3:**
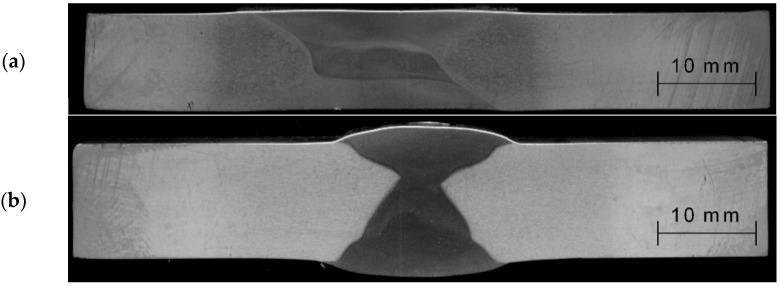
Macrostructure of the joint welded by (**a**) FSW and (**b**) MIG.

Joints welded by both methods have been verified by X-ray flaw detection and showed no welding defects. Cross-sectional views of the joints are shown in Figure 3.

**Figure 4:**
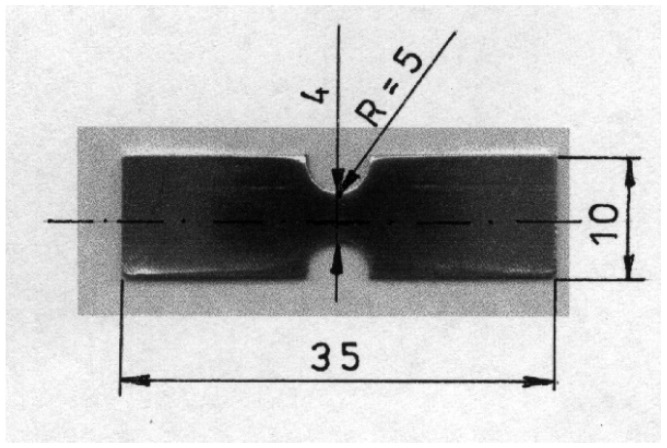
The shape and dimensions of a flat sample for an in situ tensile test in SEM.

The first stage of the research consisted of the observation of changes on the surface of the deformed sample in the vacuum chamber of a Philips XL30 scanning electron microscope (Amsterdam, Netherlands). The Microtest strain gauge from DEBEN (“in-situ tensile test in SEM”) was used for the tests. It is a tool for testing controlled deformation of samples, current recording of stress characteristics appearing in samples, and simultaneous observation of changes in the microstructure of the tested material. Samples with a thickness of 1 mm and a cross-section of F = 4 mm^2^ were used for the tests. The observed surfaces of the sample were polished. The dimensions and shape of the sample are shown in Figure 4.

The cross-sectional area of the sample was selected depending on the tensile strength of the tested material. The samples were stretched at a speed of έ = 0.05 mm/min until destruction, while observing changes in the microstructure of the tested materials in the electron beam.

It allows us to record images from the scanning microscope during stretching or when the feed of the holders was stopped.

In order to precisely determine the place and method of crack initiation, interfacial boundaries (precipitations) on plastically deformed samples were examined. Samples were lightly etched with Keller’s reagent to reveal precipitates. A chemical analysis of selected precipitates was performed by energy dispersive spectrometry (EDS) using the EDAX adapter, with background correction.

The second stage of the research was carried out using slow strain rate testing (SSRT) in accordance with ISO 7539-7:2005. The tests were carried out on a test stand that allowed a relative stretching speed of 10^−3^ to 10^−7^ s^−1^. The FT-5307 strain gauge force sensor with a measuring range of 0–16 kN was used to measure the force value, while the PSz 20 transducer with a measuring range of 0–20 mm and maximum non-linearity E = 0.159% for x = 10.00 mm was used to measure the deformation.

**Figure 5:**
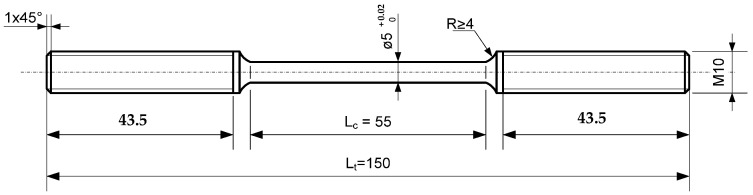
Shape and dimensions of stress corrosion test specimens.

The test consisted of increasingly deforming the specimens with the recommended strain rate for aluminum alloys έ = 1.6 × 10^−6^ s^−1^ until the complete destruction of the specimens. Smooth, cylindrical specimens with a diameter of 5 mm and an initial measuring length of L_0_ = 50 mm were used. The specimens were cut in a direction perpendicular to the rolling direction. The shape and dimensions of the specimens are shown in Figure 5.

The fracture surfaces of the specimens after the SSRT tests were subjected to fractographic analysis. Fracture topography studies were performed in order to determine the causes, nature, and course of cracking of the material under the influence of stress. They consisted of the observation of the specimens’ surface, formed as a result of the stresses leading to the separation of the material. This allowed, among others, for us to know the nature of the cracking process (ductile, trans-crystalline, cleavage, fatigue, etc.). Since the crack usually develops in the most weakened areas of the sample, various structural details could be revealed at the fracture, e.g., precipitation, material defects in the form of pores, voids, and micro-cracks.

The tests were performed using a Philips XL-30 scanning electron microscope (SEM) equipped with a tungsten electron gun. The research was carried out in a high vacuum using an SE (secondary electron) detector dedicated to determining the surface topography.

## 3. Results

The first stage of the research consisted of the observation of changes on the specimen’s surfaces deformed in the vacuum chamber of the scanning electron microscope in real-time (in situ tensile test in SEM). In the process of the destruction of the tested materials deformed at low speed, the following stages can be distinguished:

- Plastic deformations;
- The formation of micro-voids and micro-cracks;
- Joining micro-cracks and micro-voids;
- Crack propagation until material failure.

**Formation of plastic deformations**

Slip lines are formed on the surface of the plastically deformed sample, which may form slip bands as the deformation increases.

**Figure 6:**
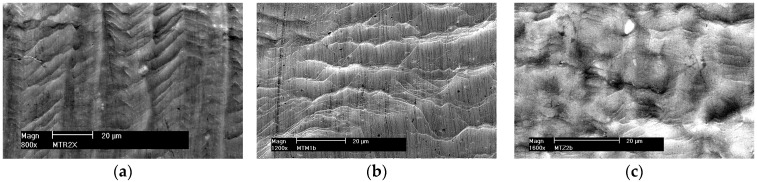
Set of plastic deformations on the surface of a sample made of alloy 5059. (**a**) Native material, (**b**) MIG weld, and (**c**) FSW weld.

Figure 6a shows the arrangement of slip bands on the surface of a polished sample made of alloy 5059. Regular interlocking slip bands are visible and arranged at an angle to each other in a system of parallel planes, suggesting that slip occurs simultaneously in different systems. The slip lines, which are up to 20 μm long, run at an angle of about 45° to the load axis in the direction of the greatest tangential stresses. Figure 6b shows a fragment of the surface of the MIG welded sample with continuous, wavy slip lines, while Figure 6c shows irregularly distributed slip lines in alloy 5059 welded by the FSW method. The exact same character of surface changes of samples during plastic deformation was observed for alloy 5083 and its joints welded by both methods: MIG and FSW (not shown in the paper).

The presented examples show a great diversity in the structure and course of the slip lines. The images of the slip lines formed in wrought aluminum alloys (Figure 6a,c) differ from those obtained in the cast weld material (Figure 6b), which proves the existence of different ways of dislocation slippage depending on the structure of the material.

**Formation of micro-voids and micro-cracks**

**Figure 7:**
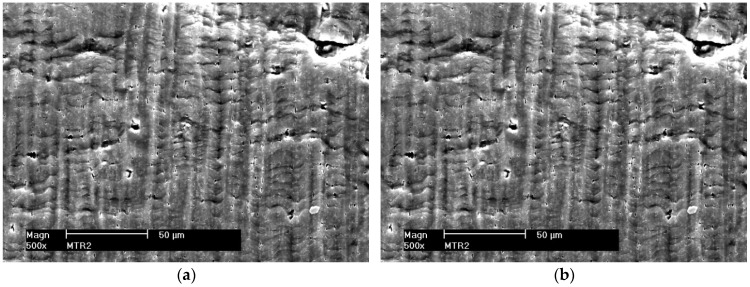
Surface fragments of deformed samples of base materials. (**a**) Alloy 5059 and (**b**) alloy 5083.

**Figure 8:**
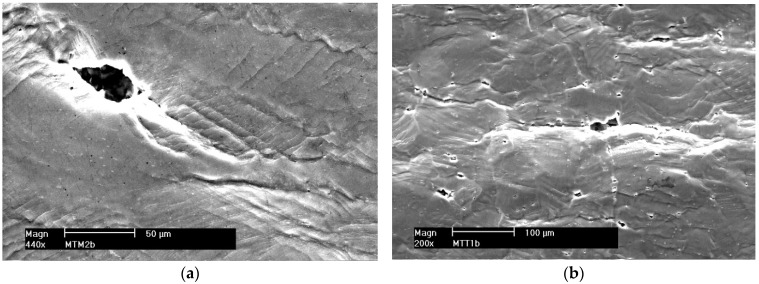
Surface fragments of deformed MIG welded samples. (**a**) Alloy 5059 and (**b**) alloy 5083.

**Figure 9:**
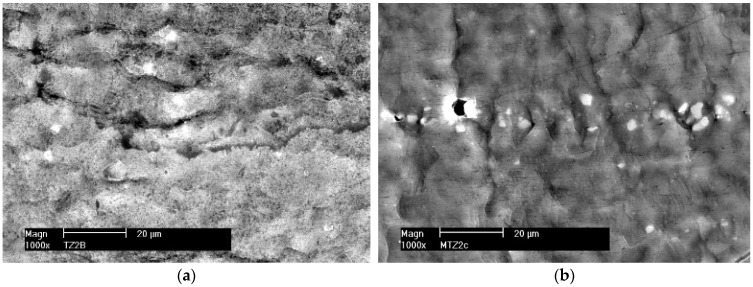
Surface fragments of deformed FSW welded samples. (**a**) Alloy 5059 and (**b**) alloy 5083.

As a result of increasing plastic deformation, micro-voids are formed in tested materials, from which micro-cracks nucleate. The resulting micro-voids were observed both in deformed alloys 5059 and 5083, as well as in their welded joints. Examples of the obtained images of the non-etched sample surface with visible micro-voids are shown in Figure 7, Figure 8 and Figure 9.

The analysis of the obtained results shows that the size of the micro-voids for native materials of the considered alloys and their welded joints are small and occur in the range of 2–10 μm. For samples welded by the FSW method, the occurrence of a smaller number of micro-voids was observed compared to the native material. Probably, the tool plasticized the material during welding leading to their fragmentation or even removal (Figure 9a). During the FSW process, the joined material is subjected to dynamic recrystallization, which fundamentally has been influenced by heat input, strain, strain rate, etc. This additionally results in grain size reduction, which plays a vital role in determining physical and mechanical properties [23].

In the case of MIG welded joints, the size of micro-voids reaches 50 μm for both alloy 5059 and 5083.

**Figure 10:**
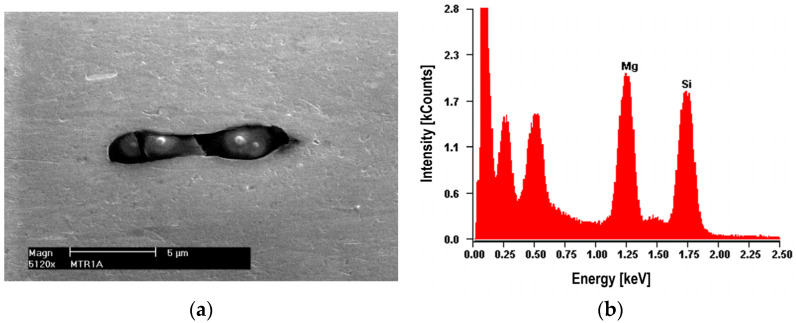
Alloy 5083. (**a**) The dark cracked precipitate of Mg_2_Si about 10 μm long with visible decohesion of interfacial boundaries along with (**b**) a microanalysis of the chemical composition of EDS.

**Figure 11:**
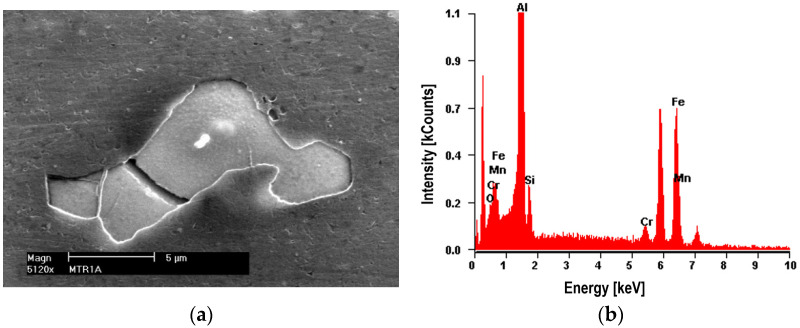
Alloy 5083. (**a**) Cracked bright precipitate with (**b**) EDS analysis. Precipitate (Mn, Fe)SiAl_12_ with a length of about 20 μm.

**Figure 12:**
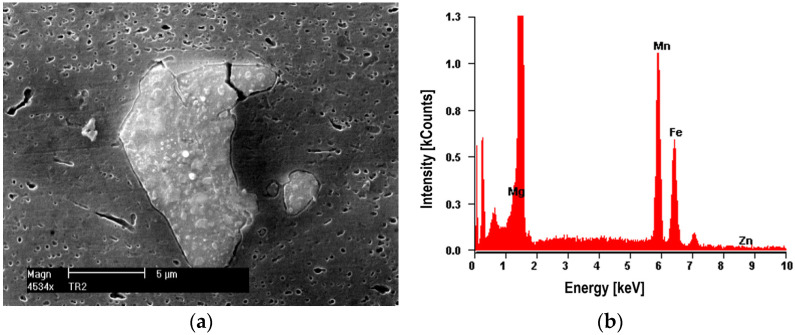
Alloy 5059. (**a**) Bright cracked precipitate of (Mn, Fe)Al_7_ with dimensions of 10 × 5 μm with (**b**) EDS analysis. Small precipitates of the Al_3_Mg_2_ phase with a diameter of up to 1 μm are visible.

**Figure 13:**
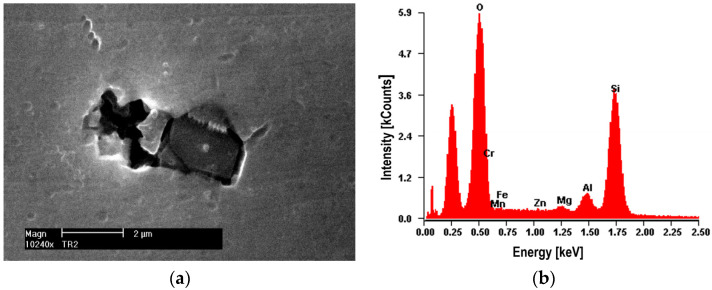
Alloy 5059. (**a**) Dark precipitate with dimensions of 2.5 × 1.25 μm (pure Si) with (**b**) EDS analysis. Visible decohesion of the matrix-molecule interface.

The nucleation of micro-voids usually occurs with the participation of precipitates. The view of sample precipitates together with their point microanalysis of chemical composition (EDS) is shown in Figure 10, Figure 11, Figure 12 and Figure 13. The places where micro-voids are formed are phase precipitates or their boundaries with the matrix. They are formed as a result of the cracking of the precipitates (Figure 10 and Figure 13) or the formation of free spaces at the interfaces between the precipitates and the matrix (Figure 11 and Figure 12). Such a mechanism occurs both in the tested Al-Mg alloys and in the welded joints.

The nucleation of micro-voids occurs with precipitates of larger dimensions, with a length of 2 to 25 μm and a width of up to 5 μm, usually of an elongated shape. The point microanalysis of the chemical composition of these precipitates shows that they contain, among others, elements such as Si and Mg or Fe, Mn, and Cr, and presumably the phases Mg_2_Si, (Fe, Mn)_3_SiAl_12_, (Fe, Mn)Al_7_, and (Fe, Mn, Cr)Al_7_ [22].

On small precipitates with a diameter of up to 1 μm, nucleation of micro-voids was not found (Figure 12). The phase equilibrium diagram of Al-Mg alloys shows that it is the β phase (Al_3_Mg_2_), strengthening the alloy.

The nucleation of micro-voids with the participation of precipitates can be explained by the different elastic and plastic properties of the precipitates compared to the matrix. According to the dislocation model of micro-void nucleation [23], dislocation loops may be formed during the deformation of alloys in the vicinity of precipitates, the blockage and accumulation of which leads to the formation of micro-voids at the interface between the matrix and the precipitates.

**Connecting micro-cracks and micro-voids**

**Figure 14:**
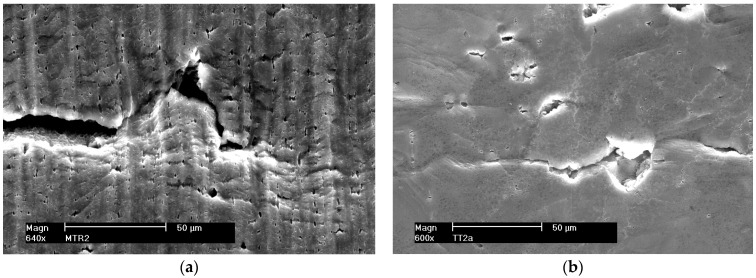
Growth of a micro-crack by connecting micro-voids; alloy 5059. (**a**) Native material and (**b**) MIG weld.

**Figure 15:**
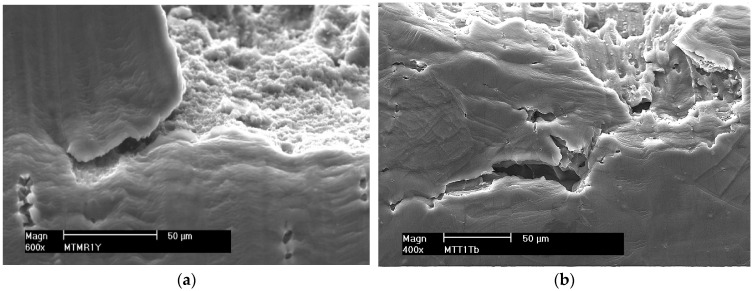
Growth of a micro-crack by connecting micro-voids; alloy 5083. (**a**) Native material and (**b**) secondary crack, MIG weld.

As a result of plastic deformation, micro-cracks and micro-voids merge. Figure 14 and Figure 15 (non-etched samples) show examples of connecting micro-cracks and micro-voids in Al-Mg alloys and their welded joints. Figure 15b shows a secondary crack branching off from the main crack.

**Crack propagation until material failure**

As the plastic deformation increases, the micro-cracks and micro-voids merge into a main crack (macro-crack). After reaching the critical dimension, the crack rapidly develops by tearing the matrix between the micro-voids, causing the destruction of the alloys.

**Figure 16:**
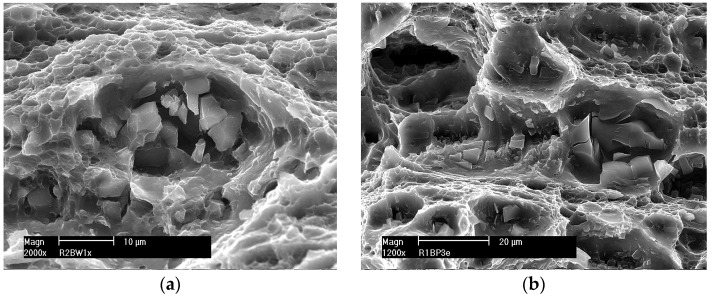
Microstructure of the crack surface with pits formed around the precipitates. MIG welded joints (**a**) 5059 and (**b**) 5083.

Figure 16 shows fragments of the surface of a ductile crack with visible dimples formed around the precipitates, which are separated by the edges of the tear. The pits visible at the fractures of the tested samples are formed on the precipitates by the nucleation of micro-voids and their growth. At the bottom of the wells, there are often precipitates of various sizes, most often destroyed.

In the destruction process of welded aluminum alloys, under the constant slow deformation conditions, ductile cracking by shear occurs, with the direction consistent with the direction of shear stress (approximately 45° to the axis of the sample). A characteristic feature of shear cracking is the presence of shear holes at fractures, which is stretched in the direction of crack propagation.

The second stage of the research consisted of stretching the samples with a slow constant strain rate (SSRT). The SSRT results, using smooth samples made of alloys 5059 and 5083 and their joints welded by the FSW and MIG methods, are presented in Table 4. The presented average values of individual parameters were calculated from three to five samples.

**Figure 17:**
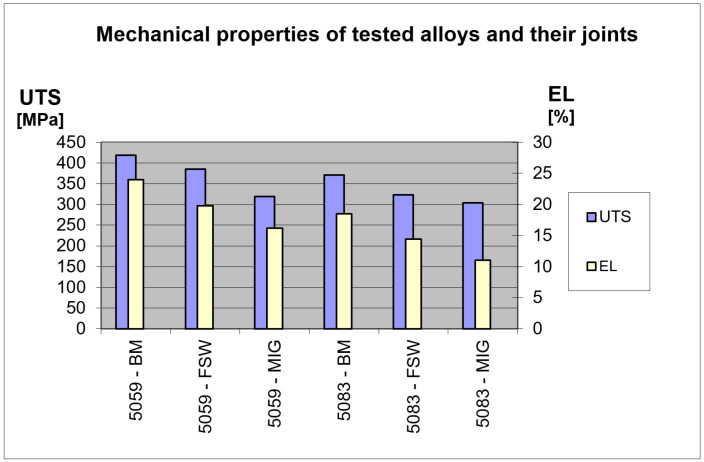
Research results of a SSRT.

For the analysis of the test results using the slow strain rate test, the two most important parameters were selected in terms of determining the mechanical properties: the maximum stress value at the moment of sample breakage, S_max_, and its elongation, EL. The graphical interpretation of the test results is shown in Figure 17.

As expected, alloy 5059 showed higher strength compared to alloy 5083. Similarly, the base material (BM) samples of both considered alloys had higher mechanical properties than their bonded joints. In the case of joints welded by the FSW method, a smaller decrease in strength and plastic properties was observed compared to the joints welded by the traditional MIG method.

Considering the effect of welding on the strength properties, it can be seen that the maximum stress value was definitely lower for welded samples compared to samples from the base material. The S_max_ parameter registered for MIG welded samples was lower by approx. 23% for alloy 5059 and 18% for alloy 5083 compared to the base material. Similar results were obtained for samples joined by the FSW method. In this case, the strength properties were also reduced compared to the base material; however, to a lesser extent. Comparing the test results, a decrease in the maximum stress of 13% for 5083 and 8% for 5059 can be seen.

Joining with both considered methods resulted in a decrease in plastic properties in relation to the native material. For MIG welded samples, the relative elongation, EL, was lower by 32% for 5059 and 40% for 5083, while for FSW welded samples, it was 17% and 22%, respectively.

**Figure 18:**
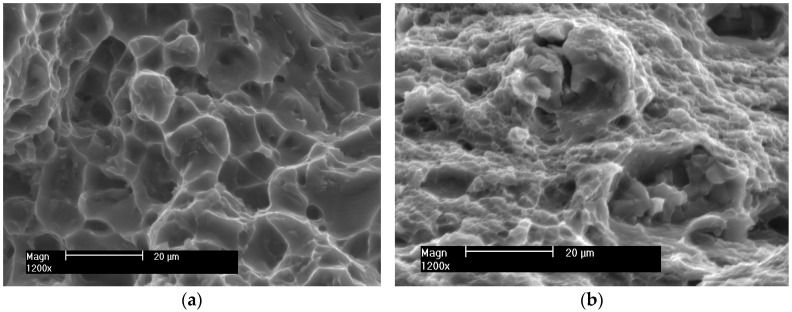
Example fractures of native material samples (**a**) 5059 and (**b**) 5083.

**Figure 19:**
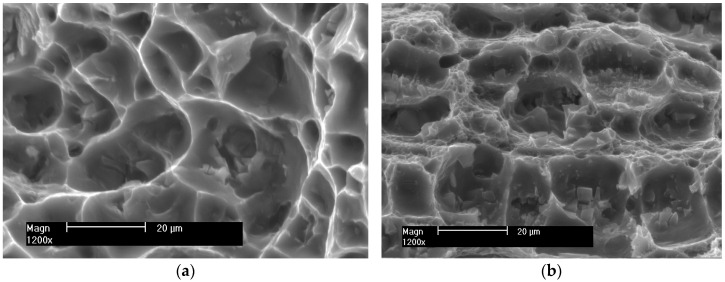
Example fractures of MIG welded samples (**a**) 5059 and (**b**) 5083.

**Figure 20:**
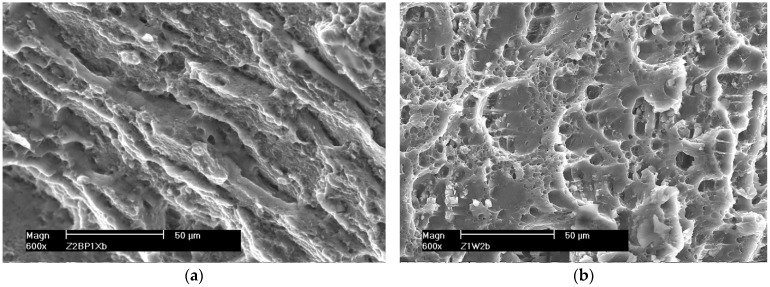
Example fractures of FSW welded samples (**a**) 5059 and (**b**) 5083.

The surfaces of the samples after the SSRT were subjected to fractographic analysis. Photographs of sample fractures of individual samples obtained with a scanning electron microscope are shown in Figure 18, Figure 19 and Figure 20.

The fractures of the samples from the native material of alloys 5083 and 5059 (Figure 18) are trans-crystalline ductile. Their surfaces are strongly developed with pits of various sizes. They are open to the effect of the increasing strain during SSRT. Micro-voids are visible, in the depth of which there are few cracked intermetallic phases.

In the case of MIG welded joints (Figure 19), the fracture is also trans-crystalline ductile. At the bottom of the wells, a significant number of cracked intermetallic phases are visible, which initiated the cracking process. This is especially evident in the case of weld 5083.

The fractures of the samples welded by the FSW method are shown in Figure 20. Micro-voids are visible on the fracture surface, but no cracked intermetallic phases exist. The cracking was ductile and mainly intercrystalline, as evidenced by the presence of a few craters open in the direction of the force loading the sample. The tearing edges are inclined in one direction. This proves that the sample cracked as a result of shearing. The crack boundaries merge into bands resulting from the impact of the plasticizing tool during the welding process.

All the tested samples were characterized by a ductile trans-crystalline fracture with a significantly developed surface. This indicates the formation of considerable plastic deformation occurring in the samples during the slow strain rate test. Cracking was initiated mainly in precipitates of intermetallic phases. The phase inclusions act as a stress concentrator necessary for the nucleation of micro-cracks. If there are no precipitates, the nucleation of micro-cracks, for the considered type of fracture, occurs in micro areas with the greatest plastic deformation [43,44].

## 4. Conclusions

The test results showed a significant influence of structural factors on the course of the destruction process of Al-Mg alloys and their bonded joints. The processes of plastic deformation, nucleation, and crack development are mainly influenced by the precipitation of intermetallic phases, heterogeneity of the microstructure, and grain size.

- Small precipitates (up to 1 μm) arranged evenly in the matrix and located close to each other can be cut by dislocations, causing plane slippage. Micro-cracks can grow along the slip line, affecting micro-cracks development. Growing, micro-cracks take the shape of a broken line, which increases the surface of the crack, and thus the energy of the cracking process increases.
- Large precipitates with dimensions from 2 μm to 25 μm, during the plastic deformation of the material, contribute to the formation of micro-voids. These precipitates occur both in native materials and in bonded joints. The results of chemical microanalysis of the precipitates (EDS) showed that they are formed on the basis of iron or silicon. Therefore, it seems advisable to strive to reduce the content of these elements in technical Al-Mg alloys.
- In base materials (5059 and 5083) and their FSW joints, evenly distributed precipitates and fine-grained structures cause the growth of micro-cracks along the broken line.
- In MIG welds, unevenly distributed precipitates in the structure and clusters of precipitates can be avoided by dislocations that bend and the slip changes its character to wavy. Micro-crack development is easier due to the coarse-grained structure and follows wavy slip lines.
- Micrographs of fractures of all investigated samples after the SSRT allow us to conclude that the cracking mechanism was trans-crystalline ductile.
- In the case of MIG welded joints, the fracture surfaces are highly developed, but there are also relatively large craters that indicate the non-uniformity of the weld structure. This inhomogeneity may be caused by the presence of grains of different sizes and intermetallic phases. These phases occur both in the form of single precipitates as well as a grid on grain boundaries. Numerous cracked intermetallic phases are visible in the wells, which probably initiated the cracking process of the material as a result of the load.
- In the case of samples from base materials of the tested alloys and their joints welded by the FSW method, smaller crater sizes were observed, which suggests smaller grain sizes compared to MIG welds. In the samples welded by the FSW method, no cracked intercrystalline phases were observed at the bottom of the wells. The crack boundaries formed parallel bands characteristic of the material deformed thermoplastically by the welding tool.
- The size of the elevations and depressions on the fracture surfaces depends mainly on the size of the grains and the dispersion of the precipitates. The void formation is easier when particle sizes are larger and the decohesion of interfacial boundaries occurs due to large local deformation around these micro-voids. As the plastic deformation increases, micro-cracks develop and merge into a major crack. After reaching a critical dimension, it develops rapidly, causing the destruction of the material.

Further research is planned on the impact of welding parameters on the grain size distribution and the size and number of both precipitates and micro-voids on the mechanisms of crack nucleation and development. It is also planned to test joints of dissimilar alloys, e.g., 5083 and 7020.

## Figures and Tables

**Table 1:** The chemical composition of the tested alloys.

Alloy	Chemical Composition [wt.%]											
	Si	Fe	Cu	Mn	Mg	Cr	Zn	Ti	Zr	B	Ni	Al
5059	0.037	0.09	0.01	0.76	5.41	0.003	0.57	0.024	0.11	0.01	0.004	bal.
5083	0.195	0.18	0.09	0.662	4.745	0.111	0.042	0.025	0.037	0.002	0.005	bal.

**Table 2:** FSW parameters of the sheets made of alloys 5059 and 5083.

Tool Dimensions			Angle of Tool Deflection	Mandrel’s Rotary Speed	Welding Speed
D [mm]	d [mm]	h [mm]	α_z_ [°]	V_n_ [rpm]	V_z_ [mm/min]
25.0	10.0	6.0	88	900	140

**Table 3:** The chemical composition of alloys used as welding wires.

Alloy	Chemical Composition [wt.%]							
	Mg	Zn	Cu	Si	Fe	Mn	Ti	Al
5383	4.0–5.2	0.4	0.4	0.25	0.25	0.8	0.15	balance
5183	4.86	0.001	0.001	0.04	0.12	0.64	0.006	balance

**Table 4:** Test results after SSRT of samples made of alloys 5059 and 5083 and their welded joints.

Alloy	Joining Method	S_max_ [MPa]	EL [%]	T_d_ [h]
5059	Native material	418.4	24.0	33.0
	FSW	384.8	19.8	28.9
	MIG	319.4	16.2	24.0
5083	Native material	371.0	18.5	26.9
	FSW	322.8	14.4	24.5
	MIG	303.6	11.0	15.8

S_max_—maximum stress value [MPa]; EL—Relative elongation [%]; T_d_—time to the destruction of the sample [h].

## Data Availability

Not applicable.
